# Genetic Risk Factors and Clinical Outcomes in Childhood Eye Cancers: A Review

**DOI:** 10.3390/genes15030276

**Published:** 2024-02-22

**Authors:** Syed Hameed, Angeli Christy Yu, Bashaer Almadani, Shereen Abualkhair, Khabir Ahmad, Giorgio Zauli

**Affiliations:** 1 Research Department, King Khaled Eye Specialist Hospital, Riyadh 11462, Saudi Arabia; bmadani@kkesh.med.sa (B.A.); sabualkhair@kkesh.med.sa (S.A.); sahmad@kkesh.med.sa (K.A.); gzauli@kkesh.med.sa (G.Z.); 2 Department of Translational Medicine, University of Ferrara, 44121 Ferrara, Italy

**Keywords:** childhood eye cancer, genetic association, common variant, rare variant, GWAS, NGS, genetic etiology, heritability, predisposition, susceptibility, survivorship

## Abstract

Childhood eye cancers, although rare, present substantial health challenges, affecting the pediatric population with a remarkable impact on their lives and families. This comprehensive review provides insights into the various types of ocular tumors, primarily focusing on malignant eye tumors, their genetic predispositions, and advancements in managing these conditions. Understanding the genetic risk factors is crucial for early detection, risk assessment, and the development of targeted therapies. This review discusses genome-wide association (GWAS) and next-generation sequencing (NGS) studies to find common and rare genetic variants. Furthermore, it also explores the outcomes and implications of these genetic discoveries in treating pediatric ocular cancer. These findings underscore the significance of genetic research in guiding early interventions and improving outcomes in children with ocular cancers.

## 1. Introduction

Childhood eye cancers are a significant health concern with far-reaching consequences for affected children and their families, influencing their physical health and quality of life. Specifically, the frequently overlooked rare and complex malignant ocular tumors require extensive investigation to elucidate their genetic foundations, clinical presentations, and adversity for the affected child’s well-being. Understanding both common and rare ocular tumors, as presented in Table 1, is vital for developing tailored treatment, early detection procedures, and advanced risk assessment tools.

Moreover, epidemiological studies providing information on the prevalence, incidence rates, and relevant demographic features further highlight the significance of these tumor conditions. The incidence of primary eye tumors in childhood is rare compared to adult tumors. For instance, in the United Kingdom, between 1963 and 2002, the annual incidence of eye tumors in children under 15 years was 3.5 per million [1]. According to the Surveillance, Epidemiology, and End Results (SEER) programme database, researchers analyzed 658 retinoblastoma cases between 1975 and 2004 and found an annual incidence rate of 11.8 per million in children under 5 years of age in the US [1,2]. Despite their rarity, pediatric ocular tumors are significantly more debilitating, causing vision impairment and blindness in early life, as well as death in the cases of ocular malignancy [3]. Pediatric eye tumors are commonly congenital with early presentation, but they may also present later in childhood [1,4]. Although benign ocular tumors are more common in children, a small percentage can develop malignant and metastatic forms [1,5]. 

In childhood, malignant eye tumors have been associated with genetic tumor predisposition syndrome [6,7]. Genetic tumor predisposition syndrome is a genetic condition caused by inherited mutations in a group of genes that increase the risk of developing certain types of tumors [6,8]. It has been suggested that inherited eye disorders are the leading cause of blindness in childhood in developed countries [9]. Therefore, the identification of ocular tumor predisposition genes is fundamental. Over the past few years, many studies have proposed the use of genetic testing, including genome-wide association [GWAS] and next-generation sequencing [NGS], to detect the genetic basis of childhood eye tumors [9,10]. The utility of these genetic tests is recognized to provide precise clinical diagnosis and improve cancer prevention, genetic counseling, and treatment options [7,9]. Therefore, the purpose of this review is to outline the various forms of pediatric malignant eye tumors and elaborate on their distinct genetic and clinical dimensions, thereby promoting an advanced understanding of these ocular cancers, better clinical management, and improved patient outcomes.

## 2. Types of Childhood Malignant Eye Cancers

### 2.1. Retinoblastoma

**Figure 1:**
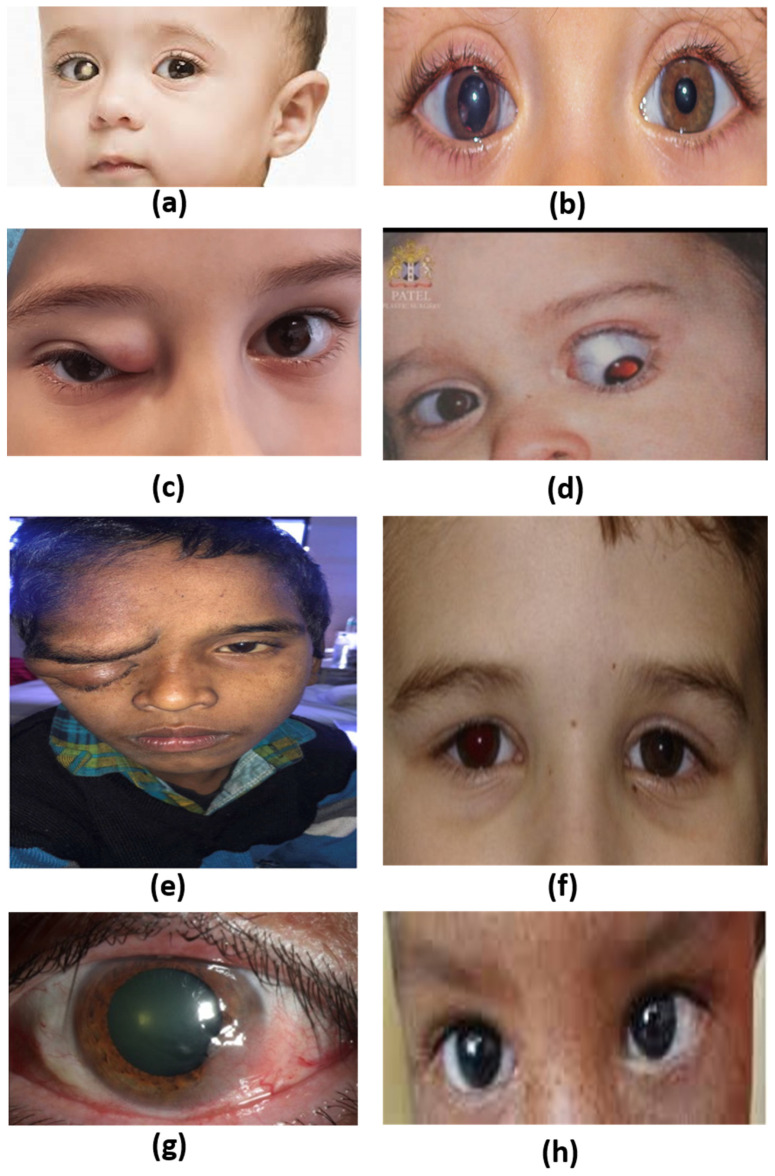
Clinical representation of childhood malignant tumors: (**a**) Retinoblastoma [19]; (**b**) Medulloepithelioma [20]; (**c**) Rhabdomyosarcoma [21]; (**d**) Optic nerve glioma [22]; (**e**) Plexiform neurofibroma [23]; (**f**) Uveal melanoma [24]; (**g**) Ocular surface squamous neoplasia [25]; (**h**) Xeroderma pigmentosa [26].

Retinoblastoma (RB) is the most common intraocular malignancy in infancy and childhood. It is an aggressive juvenile eye cancer that develops in the embryonal cells of the retina. The most frequent symptom of RB is leukocoria, which represents a white glow or white reflection of the pupil (Figure 1a). Other signs include strabismus, vision impairment, hypopyon, retinal detachment, secondary glaucoma, and ocular pain in the advanced stages [11,12]. RB can present as either a bilateral or a unilateral tumor. Sixty percent of the cases are unilateral and are diagnosed before the age of 5 years, while 40 percent are bilateral, with an early disease presentation that is usually diagnosed by 1 year of age [13,14]. For trilateral disease, retinoblastoma can develop alongside another tumor, commonly in the pineal region. Just like bilateral tumors, germline mutations promote the incidence of trilateral retinoblastoma. The average incidence of retinoblastoma is one in every 16,000 to 20,000 live births. Moreover, around 8000–9000 new cases are diagnosed each year worldwide [4,15]. Recent studies have shown that the occurrence of retinoblastoma is consistent across various demographics and is unaffected by factors such as gender, ethnicity, or socioeconomic background [14]. In terms of survival rates, 95% of patients with retinoblastoma in the United States and other high-income countries (HIC) survive, while only 30% of cases survive in low-income countries (LIC) [6,16,17]. The global survival rate for retinoblastoma is around 50%. The high survival rate in HICs stems from early detection within 14 months of age, enabling timely intervention. However, in low-income countries detection of retinoblastoma begins at 30 months. Furthermore, a systematic review explored the survival rate of RB patients in 48 less-developed countries, analyzing 164 publications and data for 14,800 patients. The findings revealed a stark income disparity, with LICs showing significantly lower rates (around 40%) compared to 77% and 79% in lower-middle and upper-middle-income countries, respectively [18]. Moreover, a study comparing survival rates by age in the US revealed that the retinoblastoma survival rate was about 99%, with an interval of 4–8 years (1994–2014) [18].

Prior investigation suggested that retinoblastoma arises from primitive retinal stem cells or cone photoreceptor cells of the retina, which exhibit a tendency towards depletion of the Retinoblastoma 1 (*RB1)* gene [27,28]. Retinoblastoma is classified into two forms: hereditary and non-hereditary (also known as sporadic or somatic) [28]. According to the two-hit hypothesis proposed by Knudson in 1971, heritable retinoblastoma is caused by one germline mutation in the *RB1* gene followed by a secondary somatic mutation that originates in retinal cells [29]. By contrast, non-heritable RB arises from two somatic mutations of the *RB1* gene acquired during retinal development [29]. While most hereditary cases of RB are bilateral, approximately 10% to 15% of children with unilateral RB are inherited in an autosomal dominant manner [14,30]. Around 80% of cases show no family history and harbor de novo germline mutations [14,30]. Children with hereditary RB or those carrying a single copy of a mutated *RB1* gene have an elevated risk of developing multiple retinoblastomas in both eyes during their early life. Moreover, they have a lifelong risk of developing non-ocular malignancies such as osteosarcomas, soft tissue sarcomas, and melanomas [6,30]. 

The *RB1* gene, responsible for retinoblastoma development, was first cloned and identified in the year 1986. The inactivation of both alleles was found to trigger the onset of retinoblastoma [15,29]. *RB1* gene is a human tumor suppressor gene that is located on chromosome 13q14.2, spans 180 kilobases [kb] of genomic DNA, and consists of 27 exons that encode for a 110 kD nuclear phosphoprotein (pRB) [15,29]. The pRB, composed of 928 amino acids, functions as a cell cycle regulator. The hypophosphorylated form of pRB is required to regulate the cell cycle checkpoints between the G1 to the S phases [15,29]. It has been suggested that pRB plays a crucial role in ensuring the proper exit of retinal progenitor cells from the cell cycle and the correct development of rod cells [13,15,29]. Thus, mutations in pRB lead to disruption of cell cycle regulation and uncontrolled cell division, causing retinoblastoma tumor development [13,15,29]. Retinoblastoma is linked to a wide variety of *RB1* gene pathogenic mutations [29,30]. In hereditary RB, nonsense (37%) or frameshift (20%) mutations within exons 2 to 25 are the most common mutations that result in the loss or inactivation of the *RB1* gene [27,29,30]. Other types of pathogenic variations that have been found to disturb the function of the *RB1* gene include point mutations, chromosomal rearrangements, large exonic deletions, and the hypermethylation of the gene promoter region [27,29,30]. Recently, it has been reported that the amplification of the *MYCN* gene may cause the development of retinoblastoma even in the absence of *RB1* mutations. It is found to occur rarely in less than 3% of children with non-heritable unilateral RB cases [27,29,30]. Additionally, MYCN-driven retinoblastomas are unilateral with an age of onset of about 4.5 months [31]. The identification of *RB1* and *MYCN* gene mutations, along with carrier screening in familial RB using NGS and/or GWAS technologies, is crucial for developing appropriate management plans for both affected children and their siblings [27]. However, it is beyond the scope of this review to discuss appropriate therapies. A management plan for children affected by these mutations should consider early enucleation.

### 2.2. Medulloepithelioma

Medulloepithelioma is a childhood malignant intraocular tumor. It is a congenital tumor originating from the primitive medullary epithelium [1,32]. It is mainly located in the non-pigmented ciliary body epithelium and can also occur in the retina, iris, and optic nerve [32]. This malignancy is formed by heteroplastic tissues, contains a network of interlaced bands of neuroepithelial cells, and resembles the neural tube [32]. Approximately at six weeks of gestation, medullary epithelial cells differentiate to form the iris, ciliary body, and retina. Medulloepithelioma tumors begin to form when the epithelial cells do not differentiate completely. The malignancy can originate as a lesion on the optic disc, iris, and retinal stalk. At early stages, medulloepithelioma has no significant effect on the function of its origin cells and thus is often not detected at an early stage. It usually becomes detectable when the tumor grows large enough to be seen through the pupil (Figure 1b). At this point, the malignancy is accompanied by symptoms such as vision loss, leukocoria, pain, and red eye [32]. Additionally, this malignancy can present as a cataract, lens subluxation, neovascular glaucoma, and cysts in the vitreous cavity from the age of 6 to 10 months. 

Although it is one of the aggressive children’s eye malignancies, comprehensive epidemiological data on medulloepithelioma is limited due to its extreme rarity. In childhood, about 78% of intraocular medulloepithelioma cases were found to be present in the first decade of life, with a median age of 2–5 years [1,32,33]. Medulloepithelioma occurs unilaterally in either eye. The condition has no gender or racial predisposition. Genetic studies show that medulloepithelioma is caused by mutations in the *DICER1* and *KMT2D* genes [32,34]. The *DICER1* gene, inherited as an autosomal dominant trait, regulates the expression of other genomes. Studies show that chromosomal alterations of the *DICER1* gene, which occur in the germ cells, cause the development of embryonic tumors, which grow to become medulloepithelioma in the eye [34]. Additionally, *KMT2D* is a tumor suppressor gene that is also related to chromosomal abnormalities associated with medulloepithelioma. Studies on the precise roles of these mutations in the development of intraocular medulloepithelioma are currently limited.

### 2.3. Rhabdomyosarcoma

Rhabdomyosarcoma (RMS) is a soft tissue tumor that can form in the orbit (Figure 1c). Among childhood orbital malignancies, RMS is the most prevalent primary tumor [35]. RMS is a primary malignant tumor that originates from pluripotent mesenchymal cells [1,36]. In childhood, RMS was found to be the most common soft-tissue sarcoma of the head and neck, accounting for 4% of all childhood malignancies and about 10% of RMS cases arising in the orbit [1,36]. Primary orbital RMS mainly affects children in their first decade of life, with approximately 70% to 90% of cases diagnosed before the age of 15 years [35,37]. It has been reported that the majority of RMS cases were diagnosed at an average age of 7 to 8 years old. However, RMS has also been found to occur at birth [35]. Children with primary orbital RMS develop a rapid onset of unilateral proptosis [36,38]. For inherited cases, the child acquires the pathogenic variant through autosomal transference. Additionally, epidemiology studies show that males are at higher risk for RMS than female children [39,40]. Additionally, adolescents are more likely to develop RMS than younger children. The malignancy can present as swelling in the eye, severe pain, and nose bleeds. It is speculated that pubertal growth increases adolescents’ susceptibility to RMS, but no empirical evidence exists to justify that. Geographically, European countries register more cases of RMS (5.4 per million children), while the United States on average reports 4.5 per million children annually [39]. 

RMS is caused by *PAX3*, *PAX7*, and *FOXO1* gene mutations [39]. The PAX3 and PAX7 are protein-coding genes and regulators of skeletal muscle cell differentiation. Similarly, FOXO1 is also a transcription factor and cell differentiation regulator. Mutations causing RMS occur when chromosomes translocate between *PAX3* or *PAX7* and *FOXO1*. Chromosomal translocation deactivates these genes, resulting in rapid growth of ocular cells that cause malignancy. The above genes are the main drivers of RMS development. In rare cases, RMS tumors can be caused by *TP53* mutation. The *TP53* gene controls the production of the p53 protein. Mutations in the *TP53* gene inhibit the formation of p53, causing uncontrolled cell division that causes RMS. Although family history with these mutations predisposes a child to develop RMS, there are no conclusive studies on the germline variants that increase the risk of cancer development [39]. Congenital orbital RMS has been associated with certain genetic predisposition syndromes. Li-Fraumeni syndrome, with germline mutations in the *TP53* gene, was noted to be correlated to primary orbital RMS [36,39]. Other syndromes that indicate a potential genetic predisposition to orbital RMS include Costello syndrome, neurofibromatosis type 1, Noonan syndrome, Gorlin syndrome, Beckwith-Wiedemann syndrome, and congenital retinoblastoma [36,39].

### 2.4. Optic Nerve Glioma

Optic nerve glioma (ONG), also known as optic pathway glioma or juvenile pilocytic astrocytoma, is a low-grade glial tumor arising and affecting the optic pathway, which involves the optic nerve, optic chiasm, and optic radiation [1,41] (Figure 1d). It constitutes 3–5% of all central nervous system tumors and 4% of orbital tumors in children [42]. It has been suggested that 90% of cases were detected in children before the age of 19, with a median age of 7 years [43]. Although the severity of this cancer is similar in both genders, 65% of cases are females [44]. Optic nerve glioma is an optic nerve tumor frequently linked to neurofibromatosis type 1 (NF1). It is, nevertheless, one of the most prevalent tumors associated with NF1, occurring in around 15–20% of people with the illness, but only 30–50% are symptomatic [6]. The *NF1* gene is an autosomal dominant trait with high penetrance such that children with parents with a pathogenic variant have a 50% likelihood of developing the condition. Children with primary ONG may exhibit an association with the genetic syndrome neurofibromatosis type 1 (NF1) that contributed to germline mutations in the *NF1* gene [45]. The development of ONG may also occur as a result of sporadic mutations. NF1-associated ONG cases are predominantly diagnosed in young children, with an average age of 4.5 years. In contrast, sporadic ONG is typically diagnosed in older children. ONG is usually asymptomatic; however, about 74% of sporadic ONG cases develop vision loss [45]. Inherited optic nerve glioma comprises half of the optic nerve malignancies and 1.5–4% of tumors developing on the eye’s orbit [44]. As the condition progresses, the tumor extends to the hypothalamus and third ventricle in the optic chiasma.

Etiologically, optic nerve glioma arises from malignant neoplasms that can be sporadic or associated with neurofibromatosis type 1 (NF1) [44]. In NF1, the mutation deactivates neurofibromin protein, a tumor suppressor, causing the growth of malignant tumors on the optic nerve. However, for sporadic cases, mutations occur when *BRAF* and *KIAA1549* fusion occurs [44]. Early detection and treatment are vital for positive patient outcomes.

Furthermore, optic nerve glioma is associated with some pathophysiological mechanisms. Usually, gliomas are benign while malignant forms are very rare. Malignant gliomas develop on the astrocytes of the optic nerve and vision pathway [44]. The tumor develops and extends to the pia, arachnoid, and subarachnoid mater, causing fibrovascular and meningeal cell proliferation which leads to tumor enlargement. Symptoms of optic nerve glioma include early vision loss, optic disc swelling, severe headaches, nausea, dizziness, developmental regression, and growth retardation [44]. Nevertheless, extensive research is necessary for developing tailored medications that improve patient outcomes.

### 2.5. Plexiform Neurofibroma

Plexiform neurofibromas are benign peripheral nerve tumors that can develop around the orbit, producing vision problems (Figure 1e). They are linked to neurofibromatosis type 1 (NF1) and affect roughly 25–30% of people with NF1 with an incident rate of approximately 1 per 2500 live births annually [23]. The condition is inheritable in an autosomal dominance pattern. Penetrance to the offspring is completed by age five, during which symptoms develop [23]. In 50% to 70% of reported cases of plexiform neurofibromas, patients have inherited the condition [23]. 

The causal agent is the mutated *NF1* gene located on chromosome 17 in locus q11.2 [23]. The *NF1* gene codes the translation of neurofibromin protein, which is a tumor suppressor. Therefore, mutations deactivate the *NF1* gene’s alleles, inhibiting neurofibromin synthesis and making a person susceptible to sporadic neurofibromas and other tumors. Despite the rarity of the cases in the pediatric population, registered cases support plexiform neurofibromas cancer in children presenting with eyelid or periorbital swelling. Additionally, children harboring this cancer exhibit learning difficulties and mental retardation.

### 2.6. Uveal Melanoma

Ocular melanoma is a malignant tumor derived from melanocytes in the uveal tract, or conjunctiva [46]. Uveal melanoma (UM) is a rare intraocular malignancy in children and young adults, with an incidence rate of less than 1% of all patients with malignant UM [46]. Uveal melanoma involves the iris, ciliary body, and choroidal melanoma [34,46] (Figure 1f). The onset of UM in children occurs around or after puberty and rarely in newborns [24,46]. A previous study in the United States involving 122 children and teenagers aged 20 years or younger with UM found that about half of the cases were aged 15–20 years. Only 3 percent of them were 5 years old or younger [24]. Another study has shown that the age-specific incidence of UM between 1973 and 1997 in the USA, per million population, for 0–4 years was 0%, 10–14 years was 0.2%, and 15–20 years was 0.4% [47]. In children with UM, the tumor was found to be located mostly in the iris [24,46]. 

In 2012, Shields et al. showed that the clinical features of UM in children are significantly different from those in adults based on 8033 cases of UM [24,46]. One of these findings suggests that the tumor originates mainly from the iris [24,46]. Iris melanoma is typically diagnosed in early life, before the age of 20. It is characterized by heterochromia and corectopia [46]. Congenital UM is extremely rare, as only six cases have been previously reported [48]. Additionally, despite the five-year survival rate after intervention being 80%, 60% of patients develop recurrent metastases which eventually cause death. 

Investigations have identified the association between the development of primary UM and constitutional pathogenic mutations in BRCA1-associated protein 1 (BAP1) [34,46]. The *BAP1* gene is a tumor suppressor gene located in chromosomal region 3p12 that is suggested to have a role in cell proliferation and growth inhibition [34]. However, the mutation causes rapid cell multiplicity, leading to metastases characterizing uveal melanoma. Approximately 50% of primary UM cases were found to harbor somatic mutations in the *BAP1* gene, but only 2 to 8% of UM cases were found to have a germline mutation in *BAP1* [34,49]. Germline mutations in *BAP1* inherited in an autosomal dominant manner, or somatic mutations were confirmed to cause BAP1-Tumor Predisposition Syndrome, which increases the risk of developing uveal melanoma and several other cancers [49]. In 34–45% of uveal melanoma cases, one of the *BAP1* alleles is displaced from chromosome 3, which deactivates this gene [46]. Genetic analysis using NGS assays is effective in detecting the presence of *BAP1* germline mutations [27]. On the other hand, 20–25% of cases of uveal melanoma result from genetic changes in the *SF3B1* gene [46]. SF3B1 (splicing factor 3b subunit 1) is a splicing gene that codes multiple proteins by removing non-coding sequences and including protein-coding ones that form the messenger ribonucleic acid (mRNA). When mutations occur, sequence insertions take place, causing incorrect splicing that degrades the mRNA, leading to malignant development. Similarly, *EIF1AX* gene mutations account for 20–25% of uveal melanomas [46]. *EIF1AX* codes the EIF1AX initiation factor, which forms the 43S preinitiation complex responsible for protein synthesis. Mutations in this gene cause amino acid deletions and substitutions, interfering with proper protein synthesis and facilitating the forming of uveal melanomas. Moreover, *GNAQ* and *GNA11* are also protein-binding genes whose mutation causes the formation of tumors. These mutations have been correlated with the occurrence of primary UM [27].

### 2.7. Ocular Surface Squamous Neoplasia

Conjunctival and corneal intraepithelial neoplasia, which can develop squamous cell carcinoma, are examples of ocular surface squamous neoplasia [6] (Figure 1g). Susceptibility to this condition’s pathogenesis is facilitated by exposure to ultraviolet (UV) radiation from sunlight, smoking, immunosuppression, genetic variants, severe injury on the ocular surface, chemical exposure, and a deficit in vitamin A [50]. Additionally, for patients with a genetic predisposition, acquiring human papillomavirus initiates the pathogenesis of ocular surface squamous neoplasia [50]. While it mostly affects adults, it can also develop in childhood. Data on the pathological mechanisms associated with this cancer show that DNA damage, deactivation of the DNA repair systems, and immunosuppression drive abnormalities in the *TP53* gene, which initiates tumor development [50]. Therefore, dysfunction of *TP53* allows cell proliferation beyond the G1-S cell cycle limit, causing tumor development on the eye’s ocular surface. Additionally, mutations in the *HGF* and *CREB* genes, which are cell growth regulators, promote the development of this malignancy. In rare cases, ocular surface squamous neoplasia cancer was driven by mutations in the *BRCA1*, *BRCA2*, *APC*, *MSH6*, *PDGFRA*, and *PTCH1* genes [50]. Just like the *TP53* gene, dysfunction in these genes due to UV radiation causes uncontrollable cell differentiation that leads to tumor development. Consequently, patients experience ocular pain, irritation, and loss of vision acuity.

### 2.8. Xeroderma Pigmentosa

Xeroderma pigmentosa is an exceedingly rare genetic condition that causes extreme sensitivity to ultraviolet (UV) light, increasing the risk of skin and eye cancer (Figure 1h). Because of its vulnerability to UV-induced damage, it frequently develops in childhood. However, because it is uncommon, precise epidemiological data are scarce. The incidence rate for xeroderma globally is approximately 1 per 250,000 live births [51]. Geographically, the incidence in the United States is 1 per million live births, 2.3 in Western Europe, 17.5 in the Middle East, and 45 in Japan [51]. 

Xeroderma pigmentosa is caused by mutations in DNA repair genes [52]. These genes include *XPA*, *XPB*, *XPC*, *XPD*, *XPE*, *XPG*, *XPF*, and *XPV*. When a person’s DNA is damaged, these genes participate in nucleotide excision repair. Therefore, children with variants for either of these gene mutations are susceptible to eye cancer after exposure to UV radiation [53]. The susceptibility arises from the inability of the child’s ocular cells to recover after exposure to UV light. Without the ability to recover, damaged cells begin to form lesions that gradually grow into tumors. Moreover, xeroderma pigmentosa is characterized by symptoms such as progressive cognitive degeneration, hearing loss, ataxia, and areflexia [51]. Due to the rarity of this malignancy, no effective treatment has been developed, but supportive measures to manage the symptoms exist.

### 2.9. Secondary Eye Cancers/Metastasis

Secondary eye cancers, resulting from metastasis of tumors from other body parts, are rare in children due to the rarity of juvenile tumor metastasis. They can affect a variety of eye structures [54]. For rare occurrences, the health implications are severe. For instance, metastasis from retinoblastoma due to treatment abandonment and noncompliance with the recommended care plan has a high mortality rate. Studies show that untreated retinoblastoma advances to systemic metastasis, and affected children die within 48 months [5]. Additionally, patients with ciliary body medulloepithelioma due to *DICER1* pathogenic variants may develop secondary malignancies such as pleuroblastoma, stromal tumors, and renal tumors, which usually develop at about 6 years of age [6]. Secondary eye cancers may develop with high mortality rates. Although there are control measures for aggressive malignancies, the rarity of the mentioned ocular cancers has brought about inadequate data crucial for developing effective treatments. 

## 3. Genetic Variants and Risk Factors in Childhood Eye Cancers

Germline variants enhance susceptibility to malignant ocular tumors in children in 8–12% of registered cases [6]. Germline variants are directly passed from the parent to the offspring through the ovum or sperm cell at inception. As the embryo develops, the variant spreads to somatic cells, causing the child to be predisposed to develop eye cancers. Genetic variants can be pathogenic or benign. The pathogenic variants directly cause one to develop a malignancy, while benign variants drive non-cancerous tumors [6]. Upon completion of penetrance, pathogenic germline variants drive genetic changes that induce the deactivation of cell-cycle regulatory genes and chromatin remodeling or cause DNA alterations that favor the development of malignancies [55]. Overall, genetic variants that elevate the risk of childhood eye cancer development require investigation into a patient’s genetic history, which provides a roadmap for correct diagnosis and treatment.

Most childhood eye cancers are rare, with low incident rates and insufficient epidemiological data. Such information insufficiency limits the ability of geneticists and ophthalmologists to collaborate in developing effective treatments for ocular cancers. Only in rare cases do ophthalmologists perform genetic testing on pediatric patients. As present in Table 2, a comprehensive panel of genes associated with eye cancersand frequent genetic testing could facilitate the identification of biomarkers for specific conditions, enabling accurate diagnosis and targeted therapy [6,56]. Consequently, patients with inherited ocular cancers can overcome vision impairment and live quality lives.

Furthermore, studies show that retinoblastoma is the most common pediatric ocular cancer with a genetic predisposition [6,57]. As aforementioned, retinoblastoma is caused by mutations in the *RB1* gene on chromosome 13. The mutation causes dysfunction in the two alleles of the *RB1* gene. Thus, the pathogenic *RB1* variant is transferred from parents to the offspring at inception. The resultant susceptibility to retinoblastoma facilitates the recurrence of these conditions, as affected children are highly likely to develop multiple intraocular malignancies at a young age and several extraocular cancers later in life. Therefore, follow-up care and control interventions are vital for survival.

Similarly, childhood intraocular cancers such as medulloepithelioma and uveal melanoma have genetic predisposition. For instance, the development of medulloepithelioma is associated mainly with mutations in the *DICER1* gene [6]. Therefore, pediatric patients inherit the pathogenic *DICER1* variant from a carrier parent. A child with a pathogenic *DICER1* variant is also predisposed to develop multiple brain tumors, renal tumors, pleuropulmonary malignancy, and sarcoma [6]. On the other hand, rare uveal melanoma is associated with the inheritance of the pathogenic *BAP1* variant [6]. Comorbidities include mesothelioma, cutaneous melanoma, and renal carcinoma. Most cases of uveal melanoma and medulloepithelioma affect patients with an underlying genetic predisposition. Survival is reliant on regular genetic testing and molecular analysis to allow timely detection and treatment of recurrent tumors for patients with genetic variants.

Environmental agents also influence cancer development. The malignant mutations occur after interactions with harmful cigarette smoke, UV radiation, and harmful chemicals [6]. Pathogenesis occurs when a genetically predisposed person acquires human papillomavirus in the case of ocular surface squamous neoplasia. Given that a child undergoes many developmental changes during the formative years, children with benign variants are likely to develop malignant eye tumors due to interaction with harmful environmental exposure. A thorough understanding of these genetic variations and risk factors is required for early diagnosis, risk assessment, and the development of tailored therapeutics, emphasizing the need for current research on this subject.

### 3.1. Genome-Wide Association Studies [GWAS]

Genome-wide association studies [GWAS] are a robust and comprehensive genetic research method for identifying genetic variations known as single nucleotide polymorphisms [SNPs] throughout the whole genome. These studies seek to discover links between specific genetic markers and a specific characteristic, illness, or condition called genomic risk loci [6]. GWAS compares the genomes of people with a certain phenotype (e.g., pediatric malignant eye tumors) against those without the illness to uncover genetic variants related to the disease’s susceptibility or progression. The results provide crucial insight into the relation between single nucleotide polymorphisms and the genomic risk loci, helpful for establishing accurate diagnosis and development of effective treatment.

### 3.2. Significance in Understanding Genetic Architecture

GWAS has major implications for understanding the genetic architecture of pediatric malignant eye tumors. Researchers can identify particular areas or genes that are statistically related to the likelihood of acquiring certain diseases by studying the genetic variations dispersed across the genome. This method enables the discovery of genetic markers that may be associated with disease susceptibility or prognosis, giving information on the underlying genetic mechanisms that contribute to the development of these cancers.

### 3.3. Notable GWAS Findings in Childhood Malignant Eye Cancers

While the number of Genome-Wide Association Studies [GWAS] on pediatric malignant eye tumors is restricted due to their rarity, several notable discoveries have emerged. GWAS have shown unique genetic variations surrounding the *RB1* gene in retinoblastoma, underlining its essential involvement in the illness and providing putative modifier genes affecting disease severity in hereditary instances. Similarly, GWAS have shed light on the genetic underpinnings of uveal melanoma, revealing SNPs relevant to melanin production and melanocyte biology [58]. Furthermore, GWAS in ocular surface squamous neoplasia may identify genetic susceptibility indicators. While further study is required, these first GWAS findings give vital insights into the genetic variables driving these uncommon cancers, opening the door to more tailored diagnostic and treatment approaches.

### 3.4. Next-Generation Sequencing [NGS] Studies

Next-generation sequencing [NGS] has emerged as a game-changing genomics tool, dramatically improving our capacity to investigate pediatric malignant eye tumors. NGS enables speedy and cost-effective DNA sequencing, allowing for complete investigation of the whole genome, including coding and non-coding regions, and detecting genetic variants with unparalleled resolution. NGS plays a critical role in interpreting the genomic landscape of pediatric malignant eye tumors by finding mutations, structural abnormalities, and other genetic variants that underlie the onset and development of these uncommon malignancies [31,58]. It provides a holistic approach that identifies the genetic basis and gives insights into their heterogeneity, prospective treatment targets, and prognostic indicators.

NGS has significantly contributed to discovering genetic variants and mutations linked to pediatric malignant eye tumors. It has revealed known and unknown genomic abnormalities [5,31]. NGS investigations, for example, have highlighted the broad mutational spectrum in retinoblastoma, including the discovery of somatic mutations in the RB1 gene and other genetic modifications that might influence disease severity and development. NGS has found recurring mutations in genes such as *GNAQ* and *GNA11* in uveal melanoma, offering information on the molecular pathways underpinning tumor growth. Furthermore, NGS enables the detection of uncommon and potentially targetable mutations, opening the path for precision medicine techniques that tailor medicines to individual patients’ distinct genetic profiles. Next-generation sequencing has transformed our capacity to examine the genetic basis of juvenile malignant eye tumors, providing unparalleled insights that promise better diagnosis, therapies, and patient outcomes.

### 3.5. Integrative Analysis of GWAS and NGS Studies

Integrative analysis combining Genome-Wide Association Studies [GWAS] and Next-Generation Sequencing [NGS] data are potent tools for unraveling the complex genetic landscape of juvenile malignant eye tumors. This synergistic technique enables researchers to combine findings from large-scale association studies with the fine-grained data afforded by NGS, resulting in a comprehensive knowledge of the genetic variables driving these uncommon cancers [59]. Researchers can build a complete picture of the genetic architecture of these conditions by combining data from GWAS, which identifies common genetic variants associated with disease susceptibility, and NGS, which uncovers rare genetic mutations and structural alterations driving cancer initiation and progression.

In practice, this multidisciplinary approach can disclose numerous important characteristics of children’s malignant eye tumors. First, it can identify clinically relevant genetic markers discovered using GWAS, such as those related to illness risk or prognosis. Simultaneously, NGS can detect uncommon mutations or genomic variants specific to particular patients, allowing for individualized treatment regimens [59]. Furthermore, integrated analyses allow researchers to investigate gene–environment interactions, illuminating how genetic disposition and environmental variables combine to impact disease development. In ocular surface squamous neoplasia, GWAS and NGS can assist in determining how genetic variations interact with variables such as UV exposure and HPV infection to affect cancer risk. Overall, combining GWAS and NGS data gives a complete and detailed insight into the genetic landscape of pediatric malignant eye tumors, opening the door to more precise diagnosis, risk assessment, and targeted therapy.

## 4. Clinical Applications and Outcomes

### 4.1. Clinical Presentation and Diagnosis

Early identification of warning signs and symptoms in children is crucial to ensure timely intervention, ophthalmic diagnosis, and prompt treatment for these eye cancers. Both benign and malignant tumors can result in vision impairment, while malignant tumors can be fatal [13]. Depending on the location of the ocular tumor, whether on the orbit, eyelids, or intra-ocular tissues, childhood malignant eye tumors may have different clinical characteristics. When an orbital tumor extends into the conjunctiva and eyelid region, it is indicative of an anterior location. Young children and infants do not often complain of vision loss, and it can be challenging to evaluate their visual acuity. Nonetheless, a few characteristics ought to alert the pediatrician to take into account the potential for an intraocular malignancy and initiate a rapid referral [52]. An older child diagnosed with an intraocular tumor may report symptoms of visual difficulties or show reduced vision on school-based visual assessments. This typically happens when a tumor involves the central retina or when there is vitreous hemorrhage, or the development of secondary cataracts. Orbital tumor diagnosis and therapy have been transformed by the advancements in computed tomography (CT) and magnetic resonance imaging (MRI) [52]. Especially in the early stages, the orbital malignancies are not feasible to detect directly. As a result, they frequently grow to a comparatively considerable size before showing symptoms. Early clinical signs of an orbital tumor may also include pain, diplopia, and conjunctival edema [52].

Retinoblastoma is diagnosed typically at the age of 1 year for bilateral cases and 2 years for unilateral cases [13]. Leukocoria is one of the most common indications and symptoms. Strabismus, impaired vision, eye redness or discomfort, and changes in the appearance of the eye may also occur in children. Proptosis and visual abnormalities can arise in instances involving the orbit or optic nerve [58]. Early detection is critical, and healthcare practitioners must be alert in detecting these symptoms, especially in young children who may not be able to express their problems. A comprehensive eye examination, imaging investigations such as ultrasonography or MRI, and commonly a biopsy or fine-needle aspiration are used to confirm the diagnosis. The most common methods for diagnosing retinoblastoma are funduscopy and ultrasound, which usually show an intraocular calcified and vascularized mass. Though 90% of cases show calcification, a computed tomography (CT) scan can help identify it; however, because of the radiation risk, this procedure is avoided. Pineal and suprasellar regions are typically imaged using magnetic resonance imaging (MRI) [12].

Intraocular medulloepitheliomas are typically larger tumors that necessitate the removal of the afflicted eye. It could be benign or cancerous. Although malignant tumors can spread locally, metastatic disease is not common [13]. Complications including cataracts and secondary glaucoma are common. Despite being cytologically malignant in about 60–90% of cases, intraocular medulloepithelioma typically only affects the surrounding tissue. Histologically, the tumor is different from retinoblastoma, but clinically, it exhibits similar signs and symptoms. Since the tumor is typically contained within the globe, enucleation is usually curative [13,52].

### 4.2. Treatment Options 

Treatment options for pediatric malignant eye tumors vary and are customized to the tumor type and stage. The most frequent malignant intraocular tumor in children, retinoblastoma has a greater than 95% survival rate with available treatment options. Retinoblastoma treatment has evolved significantly during the last 10 years. Previously, the main course of treatment was enucleation; however, the global salvage rate has improved dramatically with the development of chemotherapy, laser treatment, focused radiation, and freezing or heating treatments. Chemotherapy is used to target cancer cells throughout the body and, in some situations, can be injected directly into the eye as intra-arterial chemotherapy [58]. Currently, recommended chemotherapeutic treatments consist of etoposide, vincristine, and carboplatin. Another treatment option is radiation therapy, which includes external beam radiation and plaque radiotherapy. As orbital retinoblastoma is a curable cancer, exenteration is necessary. With the right care, the child will almost always survive if the tumor is discovered while it is still inside the globe and does not invade the optic nerve, choroid, or scleral tissue. Depending on the patient’s age, location, family history, laterality, and tumor extent, a customized course of treatment is administered. These children require lifetime monitoring for tumor recurrence, and the tumor necessitates close observation for at least 3 years after therapy [12]. More advanced tumors are treated by enucleation. Cryotherapy, laser photocoagulation, episcleral plaque brachytherapy, chemoreduction, and thermotherapy are among the treatments available for less advanced tumors [52]. Regular examinations under anesthesia are required to record disease progression or reversal and to administer local therapy. In the most severe forms of retinoblastoma, salvage rates around the globe have been reported to range from 66% to 78%. After 5 years, the absence of a local recurrence or metastatic disease is regarded as a cure [13]. Emerging targeted medicines, such as those targeting particular genetic abnormalities or pathways found via genetic testing, show promise for more accurate and less intrusive treatments that might reduce the impact on vision and quality of life.

Retinoblastoma can be treated using MDM2 inhibitors [6]. MDM2 inhibitors include nutlins such as (1, 2 & 3), which interrupt interactions between MDM2 and p53. Specifically, nutlin-3a has antitumor abilities, such as inducing apoptosis in cancerous cells. Apoptosis refers to the natural process during which cells are destroyed. Therefore, nutlin-3a treatment stimulates increased production of p53, which is a tumor suppressor, and translocates this protein to the nucleus to regulate cell growth by inducing apoptosis [60]. The induced cell death inhibits the rapid cell differentiation of retinal cells, reducing retinoblastoma. Further nutlin-3a treatment stabilizes p53 levels, which then represses apoptosis, normalizing cellular activity.

In addition to the aforementioned treatment options, recent advancements in research have demonstrated strategies to improve therapeutic outcomes and reduce associated complications. One such promising approach involves the utilization of CRISPR Cas9 gene editing technology, which enables the precise redirection of anticancer signals to target retinoblastoma cells while preserving healthy tissue [61]. This innovative technique has great potential to enhance cancer treatment efficacy by minimizing off-target effects.

Moreover, there is a growing interest in employing stem cell therapy as a viable strategy for managing advanced cases of retinoblastoma following high-dose chemotherapy. According to a 2014 systematic review and meta-analysis, the combined use of high-dose chemotherapy with stem cell transplantation (HDCT-SCT) signifies the capacity to enhance the long-term survival rates of patients with trilateral retinoblastoma, with its adoption increasing from 0 to 39% since 1995 [62]. However, this treatment regimen has notable adverse effects, primarily hematological and gastrointestinal toxicities, which require careful consideration [62]. Delayed diagnosis poses a significant risk, potentially leading to the development of a highly aggressive and metastatic disease, particularly if central nervous system metastasis occurs [62]. Furthermore, increasing medication dosages may lead to exponential tumor death rates, with bone marrow suppression emerging as the most severe side effect of high-dose chemotherapy. However, this can be alleviated by stem cell transplantation to restore depleted blood progenitor cells [62]. The survival and disease-free status observed in individuals who underwent HDCT-SCT underscores the efficacy of treatment for advanced retinoblastoma [62].

Nonetheless, survivors of retinoblastoma frequently face the challenge of secondary malignancies, with osteosarcoma being the most prevalent, necessitating attention to the environmental and genetic factors influencing the progressive incidence of secondary bone sarcoma and other malignancies [62]. Particularly in low- to middle-income countries, where delayed diagnoses and treatment initiation are common due to various factors, including familial unwillingness towards enucleation procedures, HDCT-SCT has emerged as a pivotal option for managing advanced cases, despite its substantial cost implications. In light of these complexities, the combination of stem cell therapy and high-dose chemotherapy is a promising approach to improve survival rates and reduce the likelihood of treatment-related complications in retinoblastoma patients.

In the case of rhabdomyosarcoma, the most effective treatment involves confirming the diagnosis with a biopsy and treating the condition with vincristine, cyclohexane, and adriamycin in conjunction with radiation and chemotherapy [52]. It is usually diagnosed at the age of seven and males are impacted more frequently than females [13]. Additionally, enucleation is performed to treat the most advanced tumors including uveal melanoma. If not treated promptly, it tends to spread to the liver, lungs, and other distant locations [52]. Radiotherapy combined with local tumor resection may be used for less advanced tumors.

### 4.3. Impact of Genetic Factors on Treatment Response and Outcomes

In juvenile malignant eye tumors, genetic variables significantly impact therapy responsiveness and patient outcomes. For instance, in retinoblastoma, the existence of certain mutations, such as those in the *RB1* gene, might define the severity of the illness and influence treatment recommendations. Genetic testing can also uncover possible therapeutic targets for tailored therapies, increasing the likelihood of success. Furthermore, genetic discoveries can help risk assessment for hereditary disorders, allowing for proactive care options such as unaffected eye surveillance in patients with retinoblastoma susceptibility [58]. Overall, genetic insights improve treatment accuracy and play an important role in increasing survival rates and reducing long-term problems in children with ocular malignancies.

## 5. Discussion

In this study, we investigated the complex terrain of pediatric malignant eye tumors, shedding light on their medical, genetic, and clinical characteristics. Pediatric eye cancers, such as retinoblastoma, medulloepithelioma, rhabdomyosarcoma, and others, maybe rare but have a tremendous impact. These tumors have far-reaching ramifications for vision preservation and general well-being in afflicted children, in addition to their acute health impact. Their genetic basis, caused by uncommon mutations with or without genetic risk factors, has sparked renewed interest in unraveling their molecular foundations.

The GWAS information illustrated in Table 3 and NGS data in Table 4 provides useful insight into the rare and common genetic variants for childhood eye cancers including retinoblastoma, uveal melanoma, optic nerve glioma, and plexiform neurofibroma (Table 3 and Table 4). Compared to other medical conditions, these malignancies have been extensively studied to identify genetic variations that promote their development. For instance, the GWAS studies on retinoblastoma show that it is caused by *MDM2* and *CDKN1A* mutations in addition to *RB1* mutation. Most studies on retinoblastoma have focused on *RB1* mutations that result in the deactivation of its alleles. This is evident in the table whereby the NGS studies identify four mutations that occur on different exons. The mutation on exon 11 is a deletion of the protein p.R355Nfs*6, which deactivates *RB1*. As mentioned before, the *RB1* gene encodes a protein, pRB, which suppresses tumors. Therefore, the missense mutation on exon 11 inhibits the encoding of pRB, causing uncontrolled cell differentiation. Similarly, Table 4 shows a splicing mutation of *RB1* on exon 22, implying that it causes deactivation of the *RB1* gene. The splicing mutation alters the correct translation of p.E746X, causing the deactivation of the *RB1* gene. Consequently, the RB1 gene cannot encode the tumor-suppressing protein, facilitating the development of retinoblastoma in a child’s retinal cells. Moreover, retinoblastoma can be driven by amplification of *MYCN* for a certain gain, such as 17q and 18q. Such gains impede the correct translation of proteins by causing duplication and undifferentiated cells. Such outcomes facilitate the development of retinoblastoma.

On the other hand, Table 4 shows rare somatic mutations that drive the development of retinoblastoma. From the table, it can be deduced that in rare cases, retinoblastoma can be caused by a mutation on chromosome 12 of gene *MDM2*. Gene *MDM2* encodes a tumor suppressor whose deactivation can promote the development of malignancies in the retinal cells. Similarly, *CDKN1A* is a gene vital for cell cycle regulation that ensures DNA recovery after sustaining damage. Therefore, mutations on chromosome 2 inhibit its functionality, which promotes the development of retinoblastoma.

Furthermore, the NGS and GWAS present crucial information on the pathogenic mutations that cause uveal melanoma. Table 4 shows that chromosome 3 hosts three novel genetic variants that cause uveal melanoma. The *BAP1* gene undergoes alteration on exon 17. Therefore, children with this variant are at risk of unregulated cell growth in the ocular cells, causing malignancy development. Similarly, *SF3B1* and *E1F1AX* are genetic variants that predispose a child to uveal melanoma. *SF3B1* drives a frameshift deletion on exon 15. This deletion causes an abnormality in gene *SF3B1*, making the carrier susceptible to uveal melanoma.

On the other hand, somatic mutations are localized in chromosomes 5, 6, and 15, predisposing a child to uveal melanoma. For instance, *CLPTM1L* mutation causes the forming of a C allele, which promotes a person’s vulnerability to malignancies. Although it is rare, *CLPTM1L* mutation facilitates the development of lesions on the affected child’s iris, which grow into a melanoma. *HERC2* mutation in chromosome 15 and IRF4 in chromosome 6 can also drive this outcome. These mutations occur on the somatic cells, implying they are not transferred from parent to offspring.

Moreover, NGS studies show that chromosomal alterations by *NF1* and *CDKN2A* pathogenic gene variants contribute largely to the development of optic nerve glioma. NGS studies indicate that a child predisposed to optic nerve glioma due to *NF1* and *CDKN2A* genetic variants inherits these conditions from their parents. Therefore, the transference occurs through the germ cells at inception and spreads to the somatic cells as the embryo develops. *NF1* drives a splicing mutation on chromosome 17, while *CDKN2A* facilitates a deletion on chromosome 9. These events inhibit the correct translation of some useful proteins and genes. Consequently, the host of one or multiple genetic events develops malignant tumors on the optic nerve, leading to vision loss. Conversely, similar outcomes are attainable because of alterations on chromosome five due to the *TERT* variant and chromosome 20 due to *RTEL* variations.

Further, NGS and GWAS studies provide somatic and germline variants that promote the development of plexiform neurofibroma. Table 4 shows that mutations in *NFI* and *BRAF* genes cause alterations in chromosomes 17 and 7, respectively. In both events, the chromosomal alterations occur due to deletions of crucial DNA fragments that ensure normal functionality of *NF1* and *BRAF* genes. Thus, these genes are unable to synthesize proteins that regulate cell growth. As a result, malignant tumors begin forming in the affected person’s ocular cells. Beyond inheriting these biomarkers, plexiform neurofibroma can be driven by *DPH2* and *MSH6* mutations. These genetic alterations result from a child’s interaction with harmful substances, such as UV radiation, causing eye cancers. These substances substantially damage the ocular cells’ nuclei containing the DNA material. When the child’s body cannot effectively repair the DNA, the coding sequences translating growth amino acids are altered. Consequently, rapid cell proliferation occurs and manifests as a neurofibroma.

Notably, rhabdomyosarcoma is represented in the NGS studies, but there is no information in the GWAS studies. The rarity of GWAS information stems from the heredity nature of the disease, with inadequate information on mutation events driven by environmental agents. Therefore, rhabdomyosarcoma occurs when *PAX3* fuses with *FOXO1*, altering the structure of chromosome 2. Similarly, rhabdomyosarcoma can result from fusion of *PAX7* and *FOXO1* genetic materials. These events facilitate the development of malignant tumors in the eye’s orbit. Notably, Table 3 and Table 4 do not provide GWAS and NGS studies for some conditions, such as xeroderma pigmentosa, because of the inadequacy of data. Ocular cancers in children are rare. Thus, there have not been sufficient investigations into the causative agents, resulting in a lack of effective treatment for these malignancies.

## 6. Conclusions

Genomic research has shed light on the genetic markers and processes linked to some pediatric malignant eye tumors, as evidenced by GWAS and NGS investigations. These discoveries raise the prospect of more accurate diagnoses and individualized therapies based on individual genetic profiles. The combination of GWAS and NGS data holds promise for a better understanding of these cancers, allowing for a more comprehensive approach to dissecting their genomic architecture. Genetic findings have far-reaching therapeutic consequences, improving risk assessment and allowing early identification to affect therapy decisions. Childhood malignant eye tumors are on the verge of a new age of more successful and vision-preserving treatments as tailored therapeutics continue to emerge. In conclusion, this study emphasizes the need for ongoing research and collaboration in the effort to better the lives of young children suffering from these ocular cancers.

## Figures and Tables

**Table 1:** Types of childhood malignant eye tumors, their age at onset, gender predisposition, and annual incidence *.

Malignant Tumor	Age of Onset	Predominantly Affected Gender	Number of Cases Reported Annually Worldwide
Retinoblastoma	Infancy and early childhood	No significant difference	8000–9000
Ocular medulloepithelioma	Infancy and early childhood	No significant difference	Extremely rare
Rhabdomyosarcoma	Infancy and early childhood	Male	250–350
Optic nerve glioma	Late childhood	Female	Rare
Plexiform neurofibroma	Childhood	No significant difference	Rare
Uveal melanoma	Late childhood and young adulthood	No significant difference	Rare
Ocular surface squamous neoplasia	Childhood	Male	Rare
Xeroderma pigmentosa	Childhood	No significant difference	Rare
Secondary eye cancers	Varies depending on the original cancer	Varies depending on the original cancer	Rare

* The table shows only the most common and rare malignant ocular tumors, excluding benign and other tumor types.

**Table 2:** An overview of selected genes, their functions, advantages, disadvantages, and potential impact on various ocular tumors.

Gene	Function	Advantages	Disadvantages and Potential Impact of Gene Mutations	Associated Ocular Tumor
*MDM2*	Negative regulator of p53	Inhibits p53 activity, prevents apoptosis	Overexpression leads to p53 inhibition, thereby promoting tumor growth and therapy resistance	Retinoblastoma
*CDKN1A*	Cyclin-dependent kinase inhibitor	Regulates and promotes cell cycle progression	Loss of function leads to cell cycle dysregulation, uncontrolled cell division and tumor growth	
*CCND1*	Encodes cyclin D1		Overexpression leads to increased cell proliferation	
*RB1*	Retinoblastoma protein		Loss of function leads to uncontrolled cell division	
*MYCN*	Encodes transcription factor MYCN	Regulates cell growth and proliferation	Amplification and overexpression of the gene leads to aggressive tumor growth	
*DICER1*	RNA processing	Regulates gene expression	Mutations likely disrupt normal RNA processing pathways, contributing to tumorigenesis	Ocular medulloepithelioma
*KMT2D*	Histone methylation	Suppresses tumor formation	Mutations can lead to altered gene expression profiles, contributing to tumorigenesis	
*PAX3*	Transcription factor	Involved in embryonic development	Overexpression linked to tumorigenesis	Rhabdomyosarcoma
*PAX7*		Involved in muscle development		
*FOXO1*		Regulates cell cycle and apoptosis	Overexpression linked to cell death and growth inhibition	
*TP53*	Tumor suppressor	Protects against cancer development	Mutations may lead to cancer susceptibility	
*TERT*	Telomerase activity	Maintains telomere length	Overexpression linked to cellular immortality	Optic nerve glioma
*CCDC26*	Potential involvement in cilia formation	May regulate cell signaling pathways	Dysregulation of the gene can contribute to tumorigenesis	
*CDKN2A/B*	Cyclin-dependent kinase inhibitor 2A/B	Suppresses tumor formation	Loss of function promotes cell cycle progression	
*KIAA1549*	Cellular processes	May play a role in photoreceptor function	Gene fusions have been implicated in gliomas	
*RTEL*	Regulator of telomere length	Important for telomere maintenance	Mutation may lead to telomere dysfunction and genomic instability	
*BRAF*	Serine/threonine-protein kinase B-Raf	Part of the RAS/RAF/MEK/ERK pathway	Mutations lead to cell proliferation	Optic nerve glioma Plexiform neurofibroma
*NF1*	Neurofibromin 1	Regulates RAS signaling pathway	Loss of function promotes cell proliferation	
*CDKN2A*	Cell cycle regulation	Functions as a tumor suppressor	Mutation may lead to uncontrolled cell proliferation	Plexiform neurofibroma Ocular surface squamous neoplasia
*DPH2*	Essential for diphthamide biosynthesis	Vital for protein translation	Deficiency linked to susceptibility to bacterial toxins	
*MSH6*	DNA mismatch repair	Maintains genomic stability	Mutations may lead to microsatellite instability and tumorigenesis	
*CLPTM1L*	Promotes cell survival and proliferation	Regulation of cell growth and proliferation	Dysregulation of gene expression may contribute to tumorigenesis	Uveal melanoma
*HERC2*	Regulates protein degradation	May help in maintaining cellular homeostasis	Mutation may disrupt protein degradation pathways	
*IRF4*	Transcriptional regulation	Plays a role in immune response	Overexpression linked to inflammation and autoimmunity	
*SF3B1*	Splicing factor 3B subunit 1	Involved in RNA splicing	Mutations impact RNA splicing processes and tumor progression	
*EIF1AX*	Translation initiation factor	Essential for translation initiation	Mutations contributes to altered translation initiation and tumorigenesis	
*BAP1*	Tumor suppressor	Protects against cancer development	Germline mutations associated with BAP1 cancer predisposition syndrome	
*GNAQ*	G protein subunit	Activates signaling pathways	Mutations result in activation of downstream signaling pathways, contributing to tumor development and progression.	
*GNA11*				
*HGF*	Growth factor	May promote tissue regeneration	May promote tumor growth	Ocular surface squamous neoplasia
*CREBB*	Transcriptional regulation	Plays a role in cellular response to various stimuli	Dysregulation of the gene can contribute to tumorigenesis	
*BRCA1*	DNA repair	Confers DNA repair capability	Mutations increase cancer risk	
*BRCA2*				
*APC*	Tumor suppressor	Suppresses tumor formation	Mutations lead to tumorigenesis	
*PTCH1*			Loss-of-function mutations lead to basal cell carcinoma	
*PDGFRA*	Platelet-derived growth factor receptor A	Regulates cell growth and proliferation	Mutations may influence tumor growth and response to targeted therapies	
*XPA*	Nucleotide excision repair	Facilitates DNA repair	Mutations increase cancer susceptibility	Xeroderma pigmentosa
*XPB*				
*XPC*				
*XPD*				
*XPE*				
*XPF*				
*XPG*				
*XPV*	DNA polymerase	Facilitates translesion synthesis		

**Table 3:** GWAS results showing allele frequencies for SNPs associated with childhood malignant eye tumors.

Disease	Gene	Chromosome No.	SNP ID	Variant Allele	Patients (n)	Controls (n)	OR	95%CI	*p* Value	Study Reference No.
Retinoblastoma	*MDM2*	12	rs937283	G	95	70	1.74	1.21–2.51	0.01	[63]
	*CDKN1A*	6	rs1801270	A	85	90	1.98	1.16–3.37	0.0117	[64]
	*CCND1*	6	rs1059234	T	90	95	2.15	1.23–3.76	0.0065	
Uveal melanoma	*CLPTM1L*	5	rs421284	C	*	*	1.95	1.11–3.44	-	[65]
	*CLPTM1L*	5	rs452932	C	*	*	1.91	1.10–3.30	-	
	*HERC2*	15	rs1129038	T	1142	882	0.56	0.48–0.66	5.97 × 10^−12^	[66]
	*HERC2*	15	rs12913832	G	244	882	2.43	1.79–3.29	1.13 × 10^−8^	
	*IRF4*	6	rs12203592	T	137	881	1.01	0.70–1.47	1.78 × 10^−7^	
Optic nerve glioma	*TERT*	5	rs2736100	G	*	*	1.29	1.25–1.34	-	[67]
	*CCDC26*	8	rs4295627	T	*	*	1.32	1.26–1.38	-	
	*CDKN2A/B*	9	rs4977756	T	*	*	1.26	1.22–1.31	-	
	*RTEL*	20	rs6010620	T	*	*	1.34	1.28–1.39	-	
Plexiform neurofibroma	*DPH2*	1	rs7161	C	*	*	-	-	-	[68]
	*DPH2*	1	rs4660761	G	*	*	-	-	-	
	*MSH6*	2	rs1800934	T	*	*	-	-	-	

* The authors have not provided separate case and control sample numbers but referred to a combined cohort of a total number of samples.

**Table 4:** NGS results showing genetic variants found in childhood malignant tumors.

Disease	Gene	Chromosome No.	Exon	Amino Acid Change	Allele	Cosegregation in Family	Study Reference No.
Retinoblastoma	*RB1*	13	11	p.R355Nfs*6	Heterozygous	De novo	[69]
	*RB1*	13	22	p.E746X	Heterozygous	Heterozygous mother	
	*RB1*	13	24	Splice site	Heterozygous	De novo	
	*RB1*	13	2	Splice site	Heterozygous	De novo	
	*RB1*	13	27	p.R255X	Homozygous	De novo	[70]
	*MYCN*	2	-	Amplification	Amplification	*	[71]
Uveal melanoma	*BAP1*	3	17	nBAP1	Heterozygous	*	[72]
	*SF3B1*	3	15	p.Lys653_Ser657del	Heterozygous	*	
	*EIF1AX*	3	9	p.Arg14_Gly15del	Heterozygous	*	
Optic nerve glioma	*NF1*	17	4	p.R1276*	Heterozygous	*	[73]
	*CDKN2A*	9	4	Homozygous deletion	Homozygous	*	
Rhabdomyosarcoma	*PAX3::FOXO1*	2	-	PAX3::FOXO1 Fusion	Heterozygous	*	[74]
	*PAX7::FOXO1*	1	-	PAX7::FOXO1 Fusion	Heterozygous	*	
Plexiform neurofibroma	*NF1*	17	5	p.Val166fs	Heterozygous	Heterozygous	[75]
	*NF1*	17	28	p.Glu1266Ter	Heterozygous	Heterozygous	
	*NF1*	17	39	p.Phe1884Cys	Heterozygous	Heterozygous	
	*BRAF*	7	13	p.Pro25Leu	Heterozygous	Heterozygous	

* The authors have not provided a clear elucidation of the cosegregation in families with the observed genetic variants.
